# Editorial: Probiotics in aquaculture: enhancing health and sustainability

**DOI:** 10.3389/fmicb.2026.1864753

**Published:** 2026-05-20

**Authors:** Nguyen Dinh-Hung, Haitham Mohammed, Nguyen Vu Linh, Ngoc Tuan Tran

**Affiliations:** 1 Aquaculture Pathology Laboratory, School of Animal and Comparative Biomedical Sciences, The University of Arizona, Tucson, AZ, United States; 2 Department of Rangeland, Wildlife and Fisheries Management, Texas A&M University, College Station, TX, United States; 3 Department of Animal and Aquatic Sciences, Faculty of Agriculture, Chiang Mai University, Chiang Mai, Thailand; 4 Guangdong Provincial Key Laboratory of Marine Biology, Shantou University, Shantou, China

**Keywords:** antibiotic alternatives, environmental impact, gut microbiota, microbiome engineering, pathogen inhibition, sustainable aquaculture

Aquaculture has become indispensable to global food and nutritional security (FAO, 2024), but its continued expansion has intensified persistent biological and environmental pressures. Infectious diseases, however, deteriorating water quality, metabolic disorders, impaired mucosal health, and antimicrobial resistance remain major constraints to aquaculture productivity and sustainability. These challenges cannot be addressed solely through reactive disease treatment; they require preventive strategies that strengthen host resilience, stabilize microbial ecosystems, and reduce dependence on antibiotics. Probiotics have therefore evolved from being viewed as simple dietary supplements to being recognized as functional microbial tools for managing health at the interface between the host, feed, pathogens, and culture environment (Dinh-Hung et al., 2026).

This Research Topic focuses on the role of probiotics in enhancing health in aquaculture, as probiotics serve as a viable alternative to minimize reliance on antibiotics for sustainable aquaculture. The articles assembled here reflect this broader shift in aquaculture health management. Rather than treating probiotics as isolated feed additives, they position beneficial microorganisms within a microbiome-centered framework. Aquatic animals are continuously exposed to complex microbial communities in the gut, mucosal surfaces, and surrounding water. Therefore, health and disease conditions are a result of the dynamic interactions between host immunity, balanced microbial communities, nutrient metabolism, environmental quality, and pathogen pressure. Probiotic-based strategies to improve aquatic animal health are most effective when understood within this ecological context, where microbial interventions can influence both host-associated and environmental microbial communities (Fachri et al., 2024).

Tayyab et al. provide the conceptual foundation for this perspective by reviewing microbiome engineering as a strategy for improving disease resistance in aquaculture. Their comprehensive review highlights how probiotics, prebiotics, synbiotics, post-biotics, fecal microbiota transplantation, synthetic microbial communities, multi-omics, CRISPR-based approaches, and artificial intelligence are reshaping microbial intervention strategies in aquaculture. In their review, a central message is that future progress in aquaculture probiotic formulations will be increasingly determined by precision design rather than empirical supplementation. The selection of the right bacterial strains as probiotic candidates should be based on factors such as host compatibility, colonization potential, production of functional metabolites, antimicrobial activity, immune modulation, environmental stability, and biosafety. The review also emphasizes that microbiome-based interventions must be evaluated not only for biological efficacy, but also for ecological risk, regulatory feasibility, and practical applicability on farms.

At the production-system level, Zheng et al. demonstrate how probiotics can improve shrimp culture by acting directly on the rearing environment. In *Penaeus vannamei*, the periodic application of *Bacillus licheniformis* FS051 to the culture water reduced the pH to an optimal level and significantly decreased the concentrations of ammonia nitrogen and nitrite nitrogen, as well as the numbers of *Vibrio* spp. in the later stages of cultivation. These changes were accompanied by improvements in shrimp growth indicators, including length, weight, survival rate, yield, and feed conversion ratio. High-throughput sequencing further showed that *B. licheniformis* reshaped bacterial communities in both the water and shrimp intestines, increasing microbial diversity and richness, enriching beneficial genera such as *Gemmobacter, Paracoccus*, and *Bacillus* in the water, and reducing potential pathogens such as *Flavobacterium* in the shrimp intestine. This study is particularly important because shrimp health is inseparable from water quality and microbial stability. By simultaneously improving the culture environment and host-associated microbiota, *B. licheniformis* represents a system-level microbial management approach rather than a host-limited intervention.

Zhang et al. offer another example through their study of dietary *B. velezensis* NDB in black sea bream challenged with *Aeromonas hydrophila*. Fish were fed a diet supplemented with *B. velezensis* NDB before bacterial challenge. The probiotic treatment not only improved weight gain but also alleviated infection-associated pathological signs, including gill and abdominal hemorrhage, intestinal villus deformation, and inflammatory cell infiltration. The treatment enhanced antioxidant defenses by increasing superoxide dismutase and catalase activities and reducing malondialdehyde levels. It also improved the inflammatory response, upregulating anti-inflammatory markers such as *il10* and *tgf-*β while downregulating pro-inflammatory cytokines including *il1, tnf-*α, and *ifng*. These physiological benefits were accompanied by microbial restructuring, with increased abundance of beneficial taxa (such as *Bacillus* and *Ruegeria*) and reduced abundance of opportunistic genera (such as *Aeromonas* and *Vibrio*). The study, therefore, links probiotic protection to mucosal integrity, oxidative balance, immune regulation, microbial community modulation, and direct pathogen suppression.

In another study, the contribution by Wang et al. broadens the discussion beyond infectious disease control by showing that beneficial microbes may also help regulate nutrient metabolism. In Nile tilapia, excessive dietary leucine impaired growth, increased serum total cholesterol and triglycerides, promoted hepatic lipid accumulation, and activated lipid-synthesis-related pathways, including the mTOR-SREBP1c axis. Interestingly, high leucine intake also enriched intestinal *Cetobacterium*, suggesting a microbial response to diet-associated metabolic stress. Subsequent supplementation with *C. somerae* NK01 significantly reduced serum lipid indicators, decreased hepatic lipid-droplet area, and modulated lipid-metabolism-related genes, including *IRS1, PI3K, SREBP1c, ACC*, and *FAS*. This study expands the functional scope of microbiome-based strategies by showing that beneficial bacteria may not only contribute to pathogen resistance but also maintain metabolic homeostasis under intensive feeding conditions.

A coherent theme emerging from these studies is that probiotics enhance aquaculture performance through integrated effects on environmental quality, intestinal microbial ecology, mucosal barrier function, immune regulation, oxidative balance, pathogen suppression, nutrient metabolism, and growth. Their value lies not in a single universal mechanism but in their capacity to influence biological networks that shape host resilience and production performance. This complexity also helps explain why probiotic application outcomes remain variable, as efficacy depends on several variables such as strain, host, and dosage, and is influenced by diet, life stage, pathogen exposure, water quality, and farming conditions (Rahayu et al., 2024).

Future probiotics research should therefore focus on strain selection based on optimal mechanism, dosage, and delivery method, formulation stability, host–microbiome compatibility, and long-term field validation studies. It can also focus on developing synthetic synergistic probiotics and functionally enhanced strains with improved stress tolerance, colonization efficiency, and targeted antimicrobial activity, moving beyond empirical supplementation toward precision-designed microbial interventions. To avoid unintended disturbances, it is necessary to analyze the host–microbiome–environment interactions under practical farming conditions, including intensive, recirculating, and biofloc systems, as well as assess the long-term ecological impacts on water and sediment microbiomes. Furthermore, applications of multi-omics approaches, controlled challenge models, longitudinal microbiome monitoring, and commercial-scale trials will be essential for translating promising laboratory findings into reliable aquaculture farm practices. It is equally important for responsible adoption of new probiotics to have regulatory clarity, biosafety assessment, strain traceability, and practical application protocols.

Probiotics are becoming an integral element in sustainable aquaculture because they connect microbial ecosystems with nutrition, immunity, disease prevention, and environmental management. When developed and applied with scientific precision, probiotics can support healthier cultured animals, create more resilient production systems, and promote aquaculture practices that are both productive and ecologically responsible.
